# Targeting chromosome trisomy for chromosome editing

**DOI:** 10.1038/s41598-021-97580-1

**Published:** 2021-09-10

**Authors:** Takuya Abe, Yuya Suzuki, Teppei Ikeya, Kouji Hirota

**Affiliations:** Department of Chemistry, Graduate School of Science, Tokyo Metropolitan University, Minamiosawa 1-1, Hachioji-shi, Tokyo, 192-0397 Japan

**Keywords:** Genetic engineering, Chromosomes

## Abstract

A trisomy is a type of aneuploidy characterised by an additional chromosome. The additional chromosome theoretically accepts any kind of changes since it is not necessary for cellular proliferation. This advantage led us to apply two chromosome manipulation methods to autosomal trisomy in chicken DT40 cells. We first corrected chromosome 2 trisomy to disomy by employing counter-selection markers. Upon construction of cells carrying markers targeted in one of the trisomic chromosome 2s, cells that have lost markers integrated in chromosome 2 were subsequently selected. The loss of one of the chromosome 2s had little impacts on the proliferative capacity, indicating unsubstantial role of the additional chromosome 2 in DT40 cells. We next tested large-scale truncations of chromosome 2 to make a mini-chromosome for the assessment of chromosome stability by introducing telomere repeat sequences to delete most of p-arm or q-arm of chromosome 2. The obtained cell lines had 0.7 Mb mini-chromosome, and approximately 0.2% of mini-chromosome was lost per cell division in wild-type background while the rate of chromosome loss was significantly increased by the depletion of DDX11, a cohesin regulatory protein. Collectively, our findings propose that trisomic chromosomes are good targets to make unique artificial chromosomes.

## Introduction

The recently developed genome editing technology using artificial nucleases such as CRISPR-Cas9 and TALEN has been utilized for the editing of local genomic sequence^1,2^, and this technique has contributed to understanding of the functions of individual protein-coding sequences in the last decade. Only 1–2% of genomic DNA is protein-coding sequence in mammalian cells, and the function of most non-coding sequences has not been well explored. In these non-coding regions, megabase-scale long sequences such as repetitive sequences often play critical roles as a whole. Moreover, large-scale chromosomal changes including chromosome truncation, aneuploidy and translocation frequently occurs in individual organism and such changes are associated with diseases including cancer^3,4^. In order to understand the consequence of such big chromosomal changes at cellular level, the constructions of model cell line with those chromosomal changes should be valuable. However, current genome editing technic alone is not sufficient to manipulate large-scale chromosomal DNA in laboratory.

There are two well-known methods for large-scale chromosome manipulations referred to as ‘chromosome editing’ here that include negative selection-based chromosome elimination^5^ and chromosome truncation induced by targeting telomere repeat sequences^6^. The former method selects cells having lost a target chromosome via spontaneously arising chromosome loss event using a negative selection marker such as *herpes simplex virus-thymidine kinase* (*HSV-TK*) cassette inserted into a target chromosome. This method might also select cells carrying point mutations, deletions or loss of heterozygosity (LOH) at the locus^5^. In the latter, integration of telomere repeat sequence by the transfection of telomere seeding vector causes chromosome fragmentation and a new telomere formation^6,7^. Among the fragmented chromosomes, only chromosome carrying centromere sequence can be inherited and the other fragment without centromere cannot be sustained during cell division. As a result of these processes, large truncation is induced on the target chromosome. Although these methods help chromosome editing, chromosome loss or large-scale truncations often result in cell lethality or severe growth defects in vertebrates^8,9^, and most of studies to date have applied these methods to exogenous chromosomes^7,10,11^. This is because when one copy of a whole chromosome (or a part of chromosome) is lost, deleterious mutations can become apparent due to potential mutations in the other homologous chromosome. Another possible explanation is that both of two copies of the gene are required for cellular viability to maintain high dosage of gene expression. In this study, we targeted a trisomic chromosome for chromosome editing. Trisomic chromosomes are caused by errors during cell division and stably retained if they do not adversely affect cell proliferation. Assuming one of trisomic chromosomes is dispensable for cellular proliferation, theoretically chromosome loss or large-scale truncation should be applicable to one of trisomic chromosomes. In fact, several studies have reported trisomy corrections in human cells^5,12^.

In this study, we applied two methods of chromosome editing, negative selection marker-based chromosome elimination and telomere seeding vector-mediated chromosome truncation, to the trisomic chromosome 2 in genetically amenable chicken B lymphatic DT40 cells^13^. DT40 cells are characterized by a rapid cell growth (the doubling time is about 8 h) and high gene-targeting efficiency^13^. While the karyotype of DT40 cells is stable in culture, their chromosome 2 turned to trisomy and their chromosome 24 became tetrasomy in the process of establishment^14^. However, the chromosome 2 trisomy has not been corrected to disomy in the past three decades. Thus, it is unclear whether the additional copy of chromosome 2 possessing more than 2000 genes affects the phenotypes of DT40 cells. In particular, chicken chromosome 2 contains genes encoding homologous recombination-related proteins such as XRCC2, NBS1, CTIP and TOPBP1, suggesting a possible involvement of the additional copy of chromosome 2 in the gene targeting efficiency of DT40 cells. To address these possibilities, we generated chromosome 2 disomy DT40 cells (Chr2-2) from CL18, a most widely used standard strain of DT40^15^. We also attempted large-scale trunction of trisomic chromosome 2 by integrating two telomere repeat sequences near the centromere of trisomic chromosome 2 to delete the short arm (50 Mb) and the long arm (100 Mb). The resultant cell line contained 0.7 Mb mini-chromosome. Together, our data sparks a discussion in chromosome editing methods to target trisomic chromosomes and its application for the analysis of chromosome stability.

## Result

### Correction of chromosome 2 trisomy in DT40 cells

**Figure 1:**
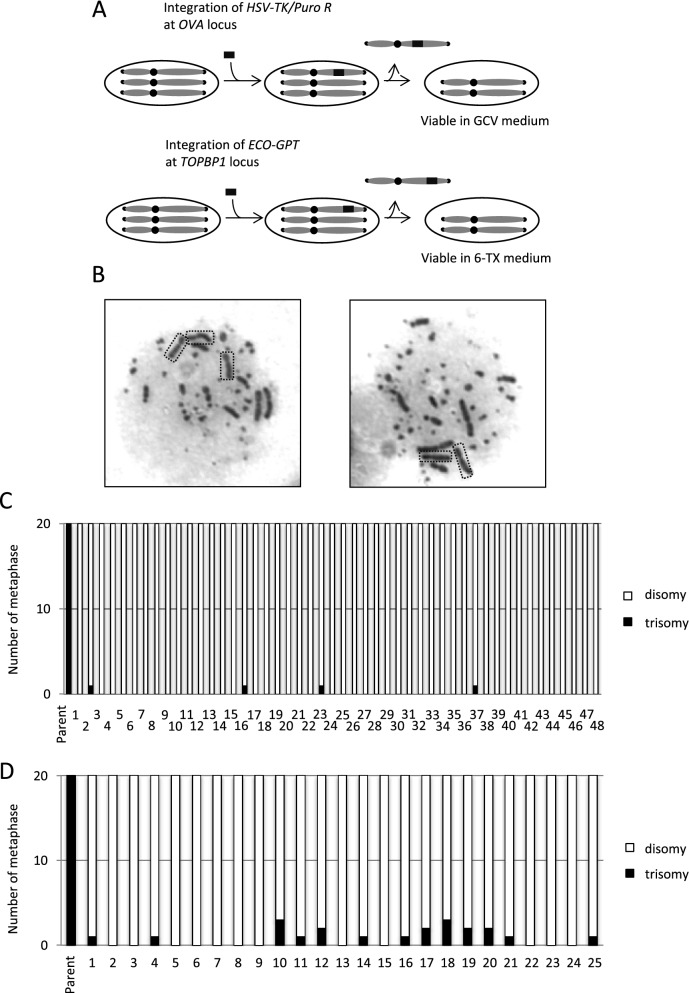
Correction of chromosome 2 trisomy in DT40 cells. (**A**) Schematic representation of trisomy correction with negative-selection markers. A negative-selection marker (*HSV-TK* or *ECO-GPT*) was integrated at *OVA* or *TOPBP1* locus on chromosome 2 by homologous recombination. After subsequent drug selection, the cells which lost the chromosome containing negative selection marker survived. (**B**) Representative images of metaphase spreads of chromosome 2 trisomy (left) and disomy cells (right). Chromosome 2s were marked by squares. See the detail of karyotype of DT40 cells in Fig. 3B. (**C**, **D**) Karyotype analyses with metaphase spreads. 48 clones were picked up from 96-well plates after GCV selection (**C**) and 25 clones were picked up after 6-TX selection (**D**).

To examine whether the additional chromosome 2 plays an important role in DT40 cells, we attempted to correct chromosome 2 trisomy to disomy. To this end, we employed a counter-selection marker, in which puromycin resistant (*puro*^*R*^) and *HSV-TK* genes are arranged in tandem, allowing us to select cells with or without this marker using puromycin or ganciclovir (GCV), respectively. This counter-selection marker was inserted at *OVA* locus on chromosome 2, widely used as a safe harbor locus in DT 40 cells^16,17^ by selecting puromycin resistant clones to generate *OVA*^+*/*+*/HSV−TK*^ cells (Fig. 1A, Supplementary Fig. S1A). The obtained *OVA*^+*/*+*/HSV−TK*^ cells were then cultured in medium containing GCV to select cells losing *HSV-TK* inserted on chromosome 2. The probability of obtaining GCV resistant clone was assumed about 0.01% (1 in 10,000). With easy identification of DT40 chromosome 2 by its shape on microscope after canonical Giemsa staining of chromosomes, the number of chromosome 2 can be effortlessly evaluated. We then counted chromosome 2 in individual 20 mitotic nuclei from 48 clones, and found only two chromosome 2s in all the clones analyzed (Fig. 1B,C). These data indicate that the chromosome 2 trisomy is corrected to disomy in these clones (hereafter referred to as DT40 Chr2-2). To investigate whether this strategy is applicable to different negative selection marker and different gene locus, we examined the similar negative selection using the *E.coli guanine phosphoribosyltransferase* (*ECO-GPT*) gene at *TOPBP1* locus on chromosome 2 (Fig. 1A). *ECO-GPT* is a useful counter-selection marker gene applicable for both positive selection (with Mycophenolic acid (MPA)) and negative selection (with 6-thioxyanthine (6-TX))^18^. Clones carrying *ECO-GPT* marker at *TOPBP1* locus were first selected with MPA, and the resultant *TOPBP1*^+*/*+*/ECO−GPT*^ clones (Supplementary Fig. S1B), then underwent negative selection using 6-TX to obtain clones that lost *ECO-GPT* marker from one of *TOPBP1*^+*/*+*/ECO−GPT*^ clones. We counted chromosome 2 in individual 20 mitotic nuclei from 25 clones, and found that the majority of all clones obtained lost one of their chromosome 2s (Fig. 1D). Small fraction of cells carrying trisomic chromosome 2 might survive because of insufficient drug selection. These results suggest that a chromosome 2 loss occurs more frequently than point/deletion mutations at *HSV-TK/ECO-GPT* gene, and that the additional chromosome 2 of DT40 CL18 cells is not essential for cell proliferation.

### Little impact of correction of chromosome 2 trisomy on cellular activity in DT40 cells

**Figure 2:**
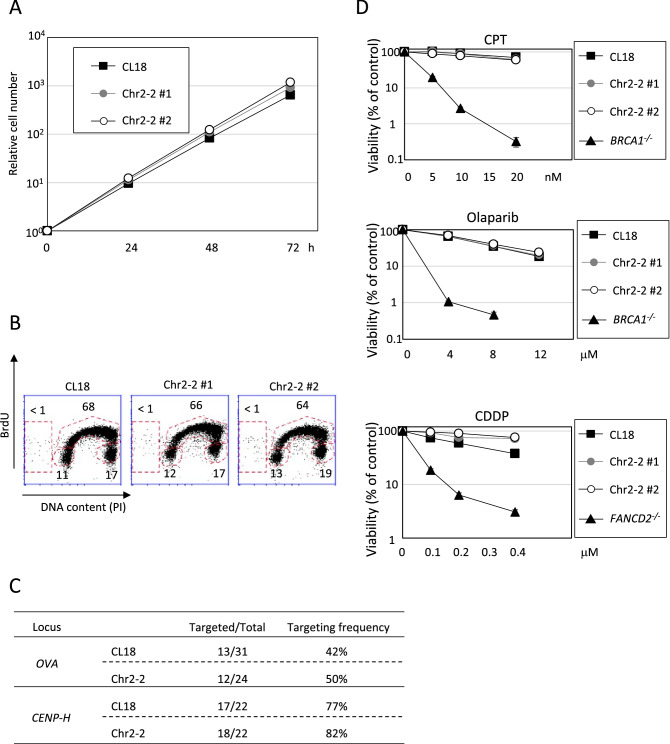
Characteristics of DT40 Chr2-2 cells. (**A**) Growth curves of DT40 Chr2-2 cells using 10^5^ cells inoculated in 1 mL of medium. Cell number was counted and passaged every 24 h. (**B**) Cell cycle distribution of DT40 Chr2-2 cells. Cells were pulse-labeled with BrdU for 15 min, harvested, and then incubated with anti-BrdU antibody. The cells were stained with Alexa488-conjugated anti-Mouse IgG co-stained with PI for the detection of DNA content. (**C**) Targeting frequency of DT40 CL18 and Chr2-2 cells. Cells were transfected with targeting constructs of the indicated loci. Targeting frequency was calculated by targeted over analyzed clone numbers. (**D**) Cellular sensitivity against DNA damaging agents. The indicated genotype cells were exposed to CPT (top), Olaparib (middle), or CDDP (bottom). The dose of the genotoxic agent is displayed on the x-axis on a linear scale, while the percent fraction of surviving cells is displayed on the y-axis on a logarithmic scale. Error bars (same size or smaller than the symbols) show the SD of the mean for 3 independent assays.

DT40 cells are characterized by the rapid cell proliferation and unique cell cycle distribution, wherein more than 60% of the cell cycle time are in the S phase. We analyzed the proliferation rate and cell cycle distribution of newly established DT40 Chr2-2 cells, and found the indistinguishable proliferation rate and cell cycle distribution between DT40 Chr2-2 and CL18 cells (Fig. 2A,B). In addition to the above-mentioned characteristics, the high efficiency of homologous recombination mediated gene targeting is the most distinctive feature of DT40 cells^13^. The gene targeting efficiency was examined at *OVA* locus^17^ and *CENP-H* locus^19^. There was similar gene targeting efficiency between DT40 Chr2-2 and CL18 cells in both loci (Fig. 2C). Similarly, DT40 Chr2-2 and CL18 cells exhibited indistinguishable cellular sensitivity against DNA damaging agents (Fig. 2D). Taken together, we conclude that loss of additional chromosome 2, containing more than 2000 genes, in DT40 CL18 cells has no effect on various characteristics.

### Establishment of trisomy derived mini-chromosome

**Figure 3:**
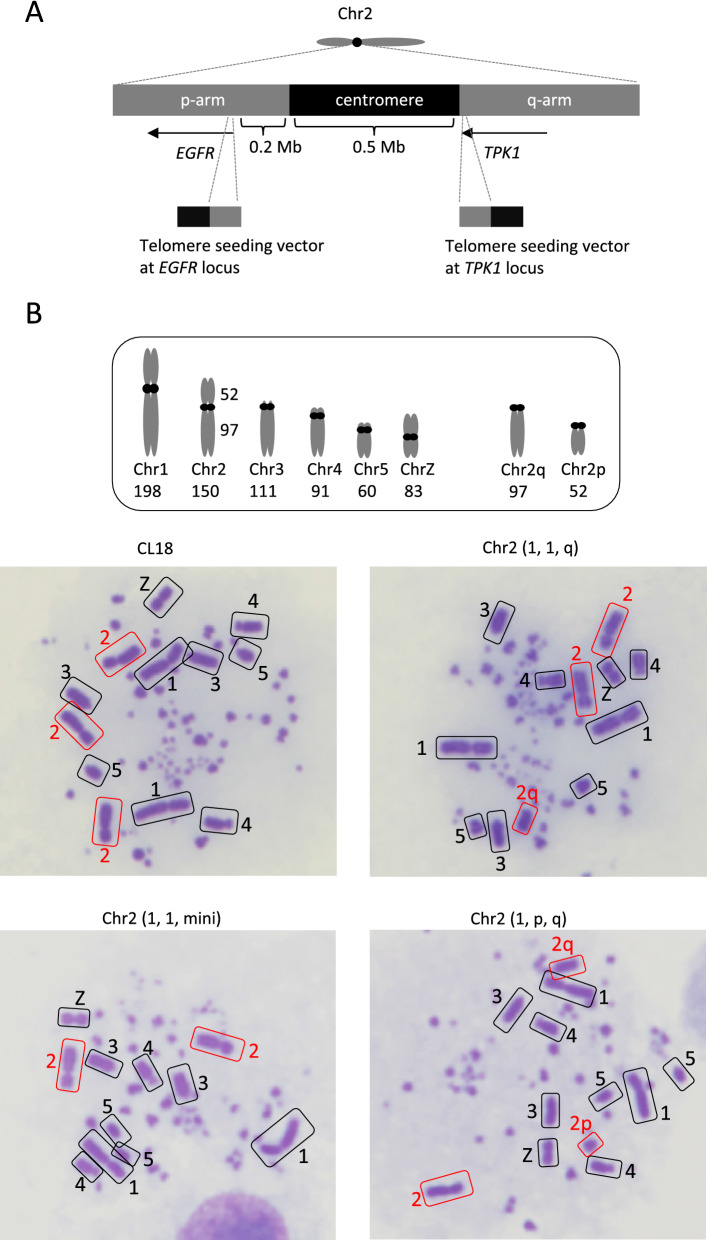
Establishment of mini-chromosome from a trisomic chromosome. (**A**) Schematic representation of the development of mini-chromosome from chromosome 2. Two telomere seeding vectors were designed at the end of *EGFR* and *TPK1* loci. The exact location of telomere seeding vectors on chromosome 2 is described. (**B**) Representative images of metaphase spreads of DT40 CL18, Chr2 (1, 1, q), Chr2 (1, 1, mini) and Chr2 (1, p, q) cells. Each macro-chromosome on metaphase spreads was classified according to the shapes and length illustrated. The uniformity of karyotypes was confirmed by analyzing at least 20 metaphase cells.

Since the additional chromosome 2 in DT40 CL18 cells turned out dispensable for cellular proliferation, various large-scale chromosome editing should be applied to one of the chromosome 2s. To utilize this advantage, we sequentially integrated telomere repeat sequences near the centromere in one of chromosome 2s to induce large-scale trunction on the chromosome 2 (Fig. 3A). First, we integrated telomere repeat sequence at the *EGFR* locus, about 0.2 Mb away from the centromere of chromosome 2 to delete one of the p-arms of chromosome 2 (~ 50 Mb). We obtained 3 clones carrying telomere sequence at *EGFR* locus out of 46 clones tested (Supplementary Fig. S2A), with two intact chromosome 2s and an additional chromosome exhibiting similar but slightly smaller than that of chromosome 3. These data suggest that one of the chromosome 2s lost the p-arm (Fig. 3B, DT40 Chr2 (1, 1, q)). Then, we attempted to integrate telomere repeat sequence in one of the DT40 Chr2 (1, 1, q) clones at the *TPK1* locus, about 10 kb away from the centromere of chromosome 2 to delete one of the q-arms of chromosome 2 (~ 100 Mb) (Fig. 3A). However, four trials amounting to 100 candidate clones (24, 24, 20, 32 /each) achieved no targeted integration (data not shown). To enhance gene-targeting efficiency, we then employed CRISPR-Cas9 system. Concomitant expression of *Cas9* and gRNA designed to cut 100 bp away from the homology region significantly increased the efficiency, and 21 clones with target-integration at *TPK1* locus out of 24 clones were obtained (Supplementary Fig. S2B). Chromosome analyses revealed two different chromosomal patterns in these 21 clones. Eleven clones maintained two intact chromosome 2s and lost the chromosome 2q unlike the parental cell line (Fig. 3B, DT40 Chr2 (1, 1, mini)). It is conceivable that the telomere targeting occurs in chromosome 2q, leading to a truncation of q-arm, and thereby resulting in the generation of invisible mini-chromosome on microscopy (Supplementary Fig. S3A). On the other hand, the remaining 10 clones had only one intact chromosome 2, and instead carried additional small chromosome corresponding to chromosome 2p in size and shape (Fig. 3B, DT40 Chr2 (1, p, q)). In this case, the telomere targeting seemed to occur in one of the intact chromosome 2s (Supplementary Fig. S3A). Similar to the DT40 Chr2-2 cells, both newly established cells proliferated in the indistinguishable kinetics in comparison to the parental clone, DT40 CL18 cells (Supplementary Fig. S3B).

### Next-generation sequencing analysis to confirm the establishment of chromosome 2-edited cells

**Figure 4:**
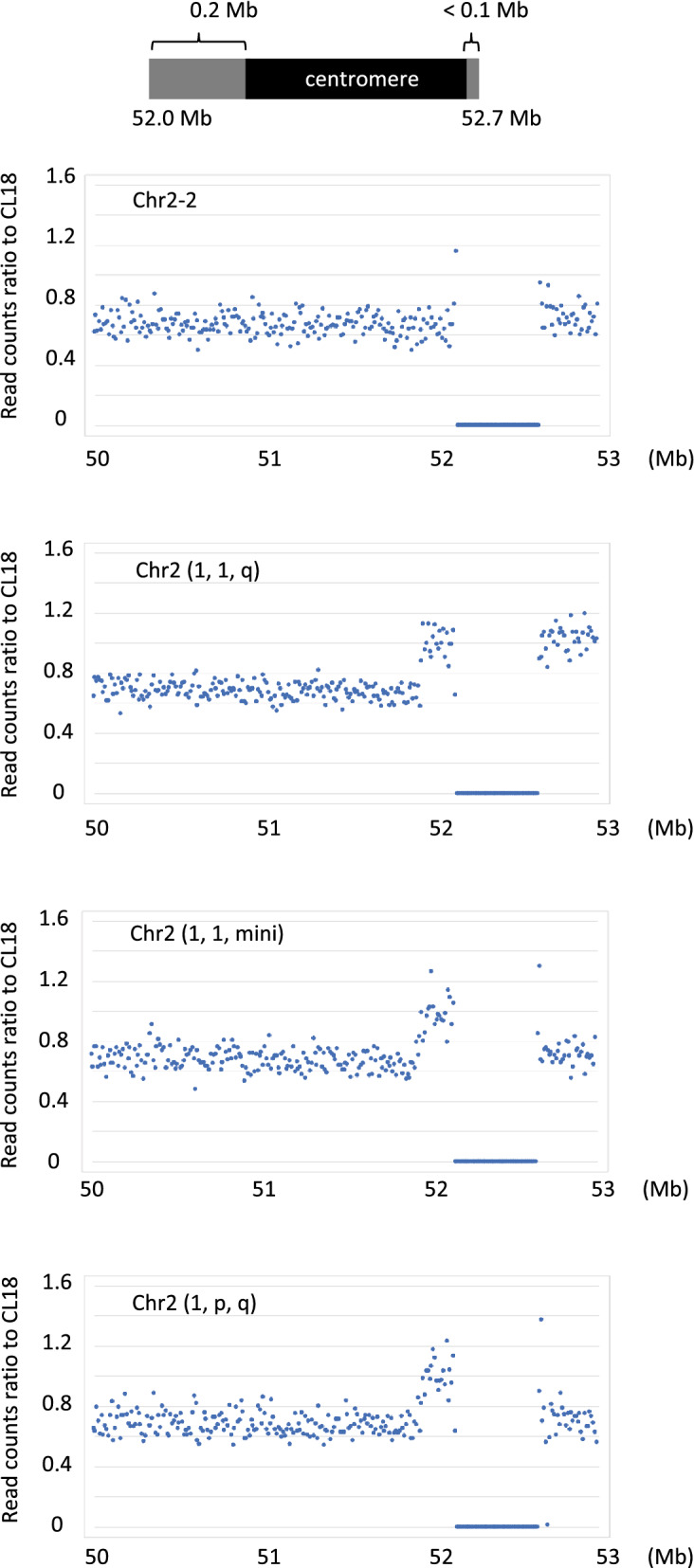
Copy number variation near the centromere of chromosome 2. The number of sequence reads mapped on the indicated positions of chromosome 2 (every 10 kb) was normalized by those of chromosome 1. This score of each strain was further normalized by that of CL18 cells. Position 52.2–52.7 Mb corresponds centromere of chromosome 2.

To confirm the establishment of chromosome 2-edited cells at genomic DNA level, we performed whole-genome sequencing with Next-generation sequencing (NGS). The copy number of each chromosome calculated by sequence read counts showed that the number of chromosome 2 decreases to two-thirds in Chr2-2, Chr2 (1, 1, mini) and Chr2 (1, p, q) cells, while the number of the other chromosomes in those cell remained unchanged (Supplementary Fig. S4A and S4B). The read counts of chromosome 2 decreased by 12% in Chr2 (1, 1, q) cells, which corresponded to the loss of a p-arm. These results indicate extensive chromosomal changes specifically happened in chromosome 2. We further analyzed the break points on chromosome 2 by examining the read counts near the centromere of chromosome 2 (Fig. 4). While the read counts of Chr2-2 cells decreased entirely in this region, Chr2 (1, 1, q) cells lost the read counts between 50 and 52 Mb region, and Chr2 (1, 1, mini) and Chr2 (1, p, q) cells further lost 52.7–53 Mb region. These data indicate that chromosome breakages specifically occurred at these points, and broken chromosomes had lost from cells.

### Monitoring loss of mini-chromosome

**Figure 5:**
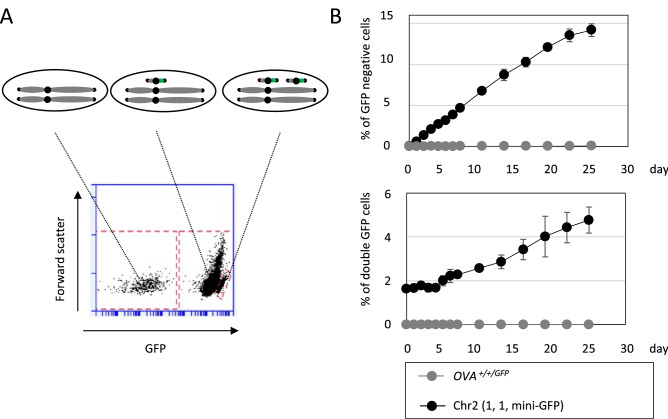
The stability of mini-chromosome. (**A**) Representative result of FACS analysis to detect the copy number of mini-chromosome. (**B**) DT40 Chr2 (1, 1, mini-GFP) and Chr2 (1, 1, 1-GFP) cells were preincubated with puromycin for 24 h to remove cells without *GFP* and *puroR* genes. Then, puromycin was washed out, and the cells were cultured for 25 days. The percentage of GFP negative and double GFP cells was analyzed by flow cytometer at indicated time points. Error bars show the SD of the mean for 3 independent cultures.

The length of mini-chromosome derived from chromosome 2 is estimated to 0.7 Mb including 0.5 Mb centromere repeat sequences^20^, and this size of chromosome is unsuited for Giemsa staining on light field microscopy. Moreover, small chromosomes, less than 1 Mb, are frequently lost during cell division^10,21^ . To understand the existence and stability of chromosome 2 derived mini-chromosome (Fig. 3A), we constructed another telomere seeding vector containing a *GFP* expression unit. Using this vector, we integrated both *GFP* gene and telomere repeat sequence at *TPK1* locus in DT40 Chr2 (1, 1, q) cells. The chromosome 2s were analyzed in 15 clones with PCR-confirmed target integration. The ratio of DT40 Chr2 (1, 1, mini-GFP) to DT40 Chr2 (1, p-GFP, q) was 8 : 7. Using a DT40 Chr2 (1, 1, mini-GFP) clone and a control cell line carrying a *GFP* gene at *OVA* locus on full-length chromosome 2, we measured the kinetics of chromosome loss by monitoring the expression of GFP by flow cytometer (Fig. 5A). Before measuring chromosome loss kinetics, cells spontaneously missing GFP labeled chromosome 2 were excluded by a positive selection, followed by measurement of rate of cells with GFP expression for 25 days. In the DT40 Chr2 (1, 1, mini-GFP) clone, approximately 0.2% of the cells lost the mini-chromosomes per division (Fig. 5B, upper panel). On the other hand, the intact chromosome 2 containing a *GFP* gene was stably inherited to daughter cells even after 25 days. We also monitored the rate of cells with double intensity of GFP signal, possibly derived from unequal mini-chromosome segregation. The population of this fraction was also increased gradually in the DT40 Chr2 (1, 1, mini-GFP) clone (Fig. 5B, lower panel). Although it is difficult to estimate the rate of unequal segregation by considering certain probability that cells with two mini-chromosomes might again lose mini-chromosome(s) during cell division, some fraction of mini-chromosome loss seems to occur as a result of unequal segregation.

### Elevated rate of mini-chromosome loss in *DDX11* deficient cells

**Figure 6:**
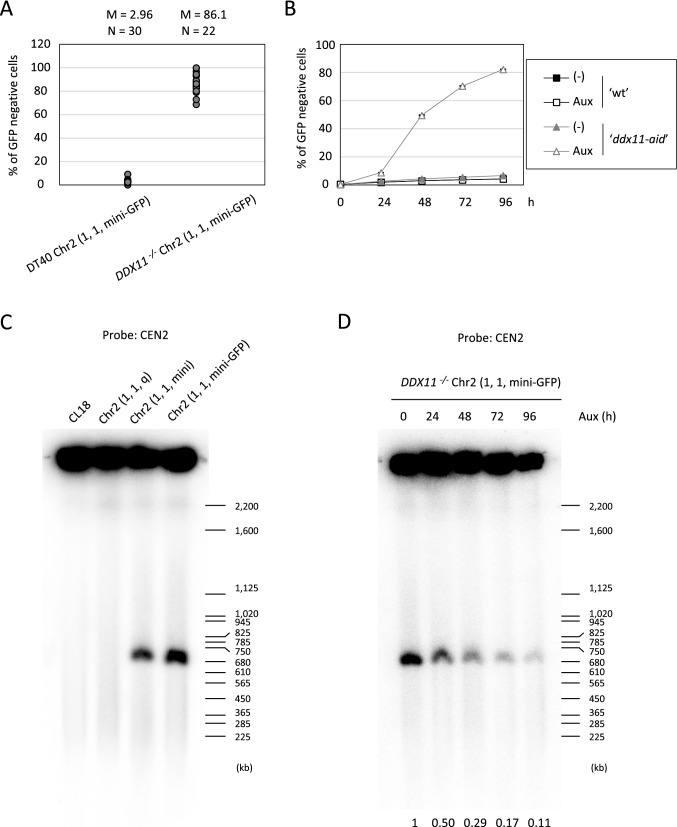
Elevated level of mini-chromosome loss in *DDX11* deficient cells. (**A**) DT40 Chr2 (1, 1, mini-GFP) and *DDX11*^*−/−*^ Chr2 (1, 1, mini-GFP) cells were preincubated with puromycin for 24 h. Then, puromycin was washed out, and the cells were inoculated in 96-well plates by limiting dilution. Each clone from single cell was analyzed by flow cytometer 7 days after the limiting dilution. The percentages of GFP negative cells were plotted by excluding clones with 100% cells losing GFP due possibly to loss of mini-chromosome at day 0 in the beginning of the growth from single cell. (**B**) DT40 Chr2 (1, 1, mini-GFP) + *TIR1-9myc* cells (shown as ‘wt’) and *DDX11*^*3AID−6FLAG/3AID−6HA*^ Chr2 (1, 1, mini-GFP) + *TIR1-9myc* cells (shown as ‘*ddx11-aid*’) were preincubated with puromycin for 24 h. Then, puromycin was washed out, and the cells were cultured for 4 days in the presence or absence of Auxin. Cells were analyzed by flow cytometer at indicated time points. Error bars show the SD of the mean for 3 independent cultures. (**C**, **D**) PFGE analyses were performed to detect chromosome 2 driven mini-chromosome. The intensity of band corresponding to mini-chromosome was normalized by that in wells. The scores below the image show the ratios to time 0.

In comparison to the similar mini-chromosome system reported previously^21^, our system showed very high rate of mini-chromosome loss (per cell division) (0.02% in Kim et al.^21^ vs 0.2% in our study). Thus, we came to consider the value of our system for the detection of chromosomal instability caused by gene mutations. To verify this system, we measured the rate of chromosome loss in *DDX11* mutant. Mutations in *CHL1/CTF1*, the yeast homolog of *DDX11*, were identified as top hit gene mutations in two independent screenings for mini-chromosome loss^22,23^ . Vertebrate’s DDX11 also plays critical roles in sister chromatid cohesion (SCC)^24–26^ , but whether *DDX11* deficiency causes increased chromosome loss has not been addressed, because of the lack of suitable experimental system. To address involvement of DDX11 in the prevention of chromosome loss, we knocked-out *DDX11* gene in DT40 Chr2 (1, 1, mini-GFP) cells as previously described^24,27^. We measured the rate of chromosome loss in DT40 Chr2 (1, 1, mini-GFP) and *DDX11*^*−/−*^ Chr2 (1, 1, mini-GFP) cells. Initially, we attempted to exclude cells spontaneously missing mini-chromosome by a positive selection as shown in Fig. 4A, but it turned out difficult to completely remove GFP negative cells in *DDX11*^*−/−*^ Chr2 (1, 1, mini-GFP) cells (discussed later). Instead, we inoculated single cells by limiting dilution and cultured from single mini-chromosome containing cell. After 7 days, flow cytometric analysis was performed with more than 22 clones from each genotype. While the median of GFP negative cells was 2.96% in DT40 Chr2 (1, 1, mini-GFP) cells, it was increased to 86.1% in *DDX11*^*−/−*^ Chr2 (1, 1, mini-GFP) cells (Fig. 6A). The estimated rates of mini-chromosome loss were 0.14%/division in DT40 Chr2 (1, 1, mini-GFP) cells and 8.98%/division in *DDX11*^*−/−*^ Chr2 (1, 1, mini-GFP) cells, respectively. Thus, DDX11 depletion increased the rate of mini-chromosome loss by about 64 times. The rate of cells with double intensity of GFP signal was also increased in *DDX11-*deficient cells (Supplementary Fig. S5). In order to exclude the possibility that augmented chromosome loss in *DDX11*^*−*/*−*^ cells was attributable to additional mutation(s) introduced during establishment of *DDX11*^*−*/*−*^ clone due to the serious chromosome instability in this clone, we conducted a similar experiment with *DDX11* conditional knockout cells. To establish *DDX11* conditional knockout cells with the auxin degron system^28^, we first expressed *TIR1-9myc* in DT40 Chr2 (1, 1, mini-GFP) cells, followed by introduction of *AID* tags to both alleles of *DDX11* genes by the Flip-in system^29^. The expression and degradation of AID-tagged DDX11 were confirmed by western blotting (Supplementary Fig. S6). Under auxin addition, the rate of mini-chromosome loss was not affected in DT40 Chr2 (1, 1, mini-GFP) + *TIR1-9myc* cells, but was accelerated to great extent in *DDX11 *^*3AID−6FLAG/3AID−6HA*^ Chr2 (1, 1, mini-GFP) + *TIR1-9myc* cells (Fig. 6B). We also explored the mini-chromosome loss event by Southern blotting. The mini-chromosome was separated from intact chromosome 2 by pulsed-field gel electrophoresis (PFGE), and detected by Southern blotting using a probe hybridized to centromere repeats of chromosome 2 (Fig. 6C, Supplementary Fig. S7A). Using this system, the acute loss of mini-chromosome in *DDX11 *^*3AID−6FLAG/3AID−6HA*^ Chr2 (1, 1, mini-GFP) + *TIR1-9myc* cells was examined. Consistent with the rates of GFP loss (Fig. 6B), we found gradual decrease in the band around 0.7 Mbp (Fig. 6D, Supplementary Fig. S7B and Fig. S7C). These results substantiated that DDX11 exerts a crucial function to prevent chromosome loss in vertebrates cells like in yeast. More importantly, our experimental system newly established is sensitive enough to detect the difference of mini-chromosome loss rate in different genetic backgrounds.

## Discussion

In this study, we performed large-scale chromosome editing to target the additional chromosome 2 in chicken DT40 cells. In contrast to normal disomy chromosomes where large-scale editing can cause cell lethality, one of the trisomy chromosomes theoretically accepts any kind of modifications if cells adapt to the resultant chromosome changes. In fact, several groups have attained trisomy correction in small human chromosomes^5,12^. However, it was unclear whether cells survive sudden loss of a copy of large chromosome, such as chicken chromosome 2 containing about 2000 genes. Thus, the successful establishment of DT40 Chr2-2 cells suggests the high adaptability of cells to change the copy number from three to two as well as the high feasibility of large-scale editing to any trisomic chromosomes.

Most studies demonstrating chromosome truncation by the integration of telomere repeat sequence in vertebrates have targeted foreign chromosomes, especially sex chromosome of other species^30–32^. The small-size mini-chromosomes derived from foreign sex chromosome and small human artificial chromosome (HAC) are known for their instability during cell division^21,31^. However, it has remained unrevealed whether the high chromosome instability just originates in their size or the difference of centromere sequence/structure between species. For these reasons, the mini-chromosome derived from an endogenous autosomal chromosome should serve as an appropriate model to answer this question. We intended to introduce a *GFP* gene into the mini-chromosome to confirm the stability of the mini-chromosome during long culture or with certain gene mutation. The rate of the mini-chromosomes loss was about 0.2% per cell division, considerably higher than similar experimental systems^21^. The fact that a 0.7 Mbp mini-chromosome containing endogenous centromere repeats is not stably inherited to daughter cells proves size of chromosome is critical for chromosome stability. It was shown that localizations of CENP-A and Aurora B which consist of kinetochore and inner centromere, respectively, are affected in mini-chromosomes^31^, indicating that certain length of chromosome might be required to fully accumulate these proteins near centromere for proper chromosome segregation.

We also demonstrated value of the system to detect chromosome loss rate in different genetic backgrounds. The current limitations of our system include that we cannot completely exclude cells missing mini-chromosome from cell culture by a positive selection, and that the rate of GFP negative fraction depends upon each cell line at the beginning of experiment. This is because several percent of GFP negative fraction still carry drug resistant genes probably due to the gene silencing of *GFP* genes in such population. One of the countermeasures for this limitation is to initiate the experiment from single cell containing a mini-chromosome (Fig. 6A). Cell sorting should provide another option to remove such GFP negative population. Another solution is to use conditional KO systems, such as auxin degron system (Fig. 6B). Though it is important to compare our system with similar experimental systems reported previously^21^, our future study should evaluate chemical substances that induce chromosome loss or genes that prevent chromosome loss.

The target integration efficiency of telomere repeat sequence is usually very low probably because it has single homology arm, and most of the related experiments have been performed in DT40 cells after transferring human chromosome to them^30,31,33^. But the development of genome editing technology allowed us to measure the efficiency in any kind of cell lines. It has already been reported that the combination of telomere seeding vector and CRISPR-Cas9 system enables target truncation of exogenous chromosome in human cells^32^. Thus, our method that changes an autosomal chromosome to a mini-chromosome maintaining only centromere and telomere (and arbitrary elements if added) is theoretically applicable to all cultured cells with trisomic chromosomes.

In this study, we demonstrated that telomere seeding vectors and negative selection markers proved powerful tools for chromosome editing. Further improvement, optimization and combination of these tools will enable modification of not only sex chromosomes and trisomy chromosomes, but also normal autosomal chromosomes to produce monosomic chromosomes or homozygous chromosome truncations. These chromosome editing options will clarify the functional significance of diploidy or highly-repeated sequences in the future.

## Materials and methods

### Cell culture

DT40 CL18 cells were kindly provided from Dr. Takeda^15^. Cells were cultured at 39.5^◦^C in D-MEM/F-12 medium (Wako) supplemented with 10% fetal bovine serum (Biowest), 2% chicken serum (Gibco), Penicillin/Streptomycin mix (Nakalai), 2 mM-L-Glutamine (Nakalai) and 10 μM 2-mercaptoethanol in the presence or absence of 500 μM of IAA (Abcam). The cell lines used in this study are listed in Table 1. To plot growth curves, each cell line was cultured in three different wells of 24-well plates and passaged every 24 h. Cell number was determined by flow cytometry using 15 μl of cell suspension, and viable cell count was determined by forward scatter and side scatter.

**Table 1:** DT40 strains used in this study.

Genotype	Plasmid (selection marker)	Reference
DT40 CL18		Buerstedde et al. 1991
*OVA*^+*/*+*/HSV−TK*^	*HSV-TK* KI at *OVA* (Puro)	This study
*OVA*^+*/*+*/GFP*^	*GFP* KI at *OVA* (Puro)	This study
DT40 Chr2-2		This study
*TOPBP1*^+*/*+*/ECO−GPT*^	*TOPBP1* KO (Eco)	This study
*BRCA1*^*−/−*^	*BRCA1* KO (Puro/His)	Martin et al., 2007
*FANCD2*^*−/−*^	*FANCD2* KO (Bsr/His)	Yamamoto et al. 2005
DT40 Chr2 (1, 1, q)	Telomere-*EGFR* (Puro)	This study
DT40 Chr2 (1, p, q)	Telomere-*EGFR* (Puro)/Telomere-*TPK1* (His)	This study
DT40 Chr2 (1, 1, mini)	Telomere-*EGFR* (Puro)/Telomere-*TPK1* (His)	This study
DT40 Chr2 (1, 1, mini-GFP)	Telomere-*EGFR* (Puro)/Telomere-*TPK1-GFP* (His)	This study
*DDX11*^*−/−*^ Chr2 (1, 1, mini-GFP)	Telomere-*EGFR* (Puro)/Telomere-*TPK1-GFP* (His)/*DDX11 KO* (Bsr/Bleo)	This study
DT40 Chr2 (1, 1, mini-GFP) + *TIR1-9myc*	Telomere-*EGFR* (Puro)/Telomere-*TPK1-GFP* (His)/*TIR1-9myc* (Neo)	This study
*DDX11*^*3AID−6FLAG/3AID−6HA*^ Chr2 (1, 1, mini-GFP) + *TIR1-9myc*	Telomere-*EGFR* (Puro)/Telomere-*TPK1-GFP* (His)/*TIR1-9myc* (Neo)/*DDX11-AID* Flip-in (Bsr/Bleo)	This study

Bleo bleomycin; Bsr blasticidin; Eco ecogpt; His histidinol; Neo neomycin; Puro puromycin.

### Plasmid construction and transfection

To knock-in *HSV-TK* gene at *OVA* locus on chromosome 2, we modified the Flip-in vector for a stable expression of cloned cDNA from β*-actin* promoter at *OVA* locus^29^. To convert the Flip-in vector to KI vector, the existing homology arm was utilized as a left arm. The Right arm of *OVA* KI vectors was amplified using the primers 5′-agtcATCGATtcccacttttcctagggaggtcttcc-3′ (ClaI) and 5′-aaagGTCGATataacttccttcctggcaatgcagtac-3′ (SalI). *HSV-TK* gene was also amplified using the primers 5′-agtcGCTAGCatggcttcgtaccccggccatcaac-3′ (NheI) and 5′-aaaaGCTAGCtcagttagcctcccccatctcccg-3′ (NheI). These amplified fragments were cut and cloned into the above vector.

*TOPBP1* KO-Eco vector was generated from genomic PCR products combined with *ECO-GPT* marker cassette. The left and right arms of *TOPBP1* KO vectors were amplified using the primers 5′-aaaaGGTACCtcctgagtaacatgtttttgtttg-3′ (KpnI) and 5′-ttttGTCGACttatgaacttcctcctgagtggag-3′ (SalI) (for the left arm of the KO construct); and 5′-aaaaGCGGCCGCcaaaaggtaaaacttatagcatacg-3′ (NotI) and 5′-ttttGAGCTCgttgttccaaaattgtattcgctaacagc-3′ (SacI) (for the right arm of the KO construct). The amplified PCR products were cut and cloned into pLoxP-Ecogpt vector^34^ using the attached restriction sites. To add 3xmAID-6xFLAG and 3xmAID-6xHA tags to DDX11 by Flip-in system, 2.3 kb upstream DNA sequence of stop codon of *DDX11* was amplified with the primers 5′-agatGTCGACttcttgtagcattagaccaagctgc-3′ (SalI) and 5′-atggACTAGTgtctgatttccctcggtggaactgg-3′ (SpeI). The amplified DNA fragment was cloned into p3xmAID-6xFLAG or p3xmAID-6xHA vector at SalI and NheI restriction enzyme sites^35^. We used pX330 vector (Addgene plasmid #42230)^36^ for the CRISPR-Cas9 system. The CRISPR expression vector for *DDX11* locus was designed to recognize 5′-gggctgatgctcgagccaat-3′. To generate telomere seeding vectors with different drug resistant markers, the telomere repeats from pSXneo 135(T2AG3) (Addgene plasmid #12402)^37^ was cloned into pLoxP vectors^34^ using NotI and SacI restriction sites. The telomere seeding vectors targeting at *EGFR* locus and *TPK1* locus were generated from genomic PCR products combined with *puroR* and *hisD* selection marker cassettes, respectively. The homology arms were amplified using the primers 5′-aaaaGCGGCCGCtgcatttaggcagaccacttcc-3′ (NotI) and 5′-aaaaACTAGTttgatgtcatgggaagccaggac-3′ (SpeI) (for *EGFR* locus); and 5′-aaaGCGGCCGCctggaaacatccgtaacttg-3′ (NotI) and 5′-aaaACTAGTaagtgctttcactgccatctcctgc-3′ (SpeI) (for *TPK1* locus). The amplified PCR products were cut and cloned into the above vectors. The *GFP* expression unit was cloned into the NheI site of the *TPK1* telomere seeding vector. The CRISPR expression vector for *TPK1* locus was designed to recognize 5′-tggatagaaaccacccacgt-3′. These vectors were transfected to DT40 cells as previously described^13^, or Neon® Transfection System (Invitrogen) according to the manufacturer's instructions. The primers used to confirm targeted integration were shown in Table 2.

**Table 2:** Primer sequences used in this study.

Primer number	Sequence
1	ggtactatgtacagcattccatcct
2	cgtataatgtatgctatacgaacgg
3	ccttaggtctgttctgccctattcagggtg
4	cttcgtataatgtatgctatacgaacggtag
5	cagcagacatgccacttcctgcagcatg
6	gaagtaatacatggggagagttccctgtg
7	cactttctcccgcttcataccactcccaag
8	caccacaacatgacattccagcaaaagg

### Karyotype analysis

Cells were treated with 0.1 μg/ml colcemid for the last 2 h to increase metaphase-arrested cells and then harvested. The harvested cells were treated with 75 mM KCl for 13 min at room temperature and fixed with methanol-acetic acid (3:1) for 30 min. The cell suspension was dropped onto ice-cold glass slides, air dried and stained with 5% Giemsa solution, pH 6.8, for 10 min, and then examined by light microscopy. All images in this study were collected with a camera (visualix) mounted on a microscope (ECLIPSE Ni; Nikon).

### Western blotting

Western blotting was performed as previously described^24^ using antibodies against FLAG (Sigma), HA (Roche) and β-actin (Proteintech) followed by horseradish peroxidase-conjugated anti-rat or anti-mouse IgG secondary antibody (Cell Signaling). Proteins were visualized using ImmunoStar® LD with manufacturer’s protocol.

### Quantification of rate of mini-chromosome loss

To calculate the rate of mini-chromosome loss per cell division, we used the equation;

$${\text{f}}_{{\text{n}}} = {\text{ f}}_{0} \left( {{1} - {\text{r}}} \right)^{{\text{n}}}$$

where n is the number of cell division; r is the rate of mini-chromosome loss per division; and f_n_ is the rate of cells retaining mini-chromosome. When the experiment starts from single cell, f_0_ is 1. DT40 cells divide three times per day. The fact that the rates of double GFP cells are much lower than those of GFP negative cells led us to ignore the generation and loss of cells containing two mini-chromosomes for simplification.

### PFGE

Agarose plugs of chromosomal DNA were prepared following the protocol described previously^38^. Electrophoresis was performed for 16 h at 14 °C through 1% agarose in Tris–borate–EDTA buffer using a CHEF-DR III apparatus with the following parameters: interval, 60–120 s; angle, 120°; voltage, 6 V/cm. After electrophoresis, the gel was stained with 0.5 μg/ml ethidium bromide for 30 min, destained in deionized water for 20 min, and photographed.

### Southern blotting

The DNA separated by PFGE was transferred to a nylon membrane (Biodyne B, PALL, NY). To detect centromere repeats of chromosome 2, a 870 bp PCR product, amplified by primer 7 and primer 8 (Table 2), was used as a template for random-primer labeling (GE Healthcare) with^32^Pα-dCTP (PerkinElmer, MA). Hybridization was performed in a buffer (1% BSA, 7% SDS, 0.5 M Na_2_HPO_4_ [pH 7.4], 1 mM EDTA) at 62 °C for 12 h, and extensively washed with wash buffer (1% SDS, 1 mM EDTA, 0.04 M Na_2_HPO_4_ [pH 7.4]). Signal was detected by a phosphor imager (FLA7000, Fuji film, Tokyo).

### Genome sequencing

Genome sequencing of CL18, Chr2-2, Chr2 (1, 1, q), Chr2 (1, p, q) and Chr2 (1, 1, mini) cells were outsourced to Macrogen using HiSeq X (Illumina). Genomic DNA were prepared using PureLink™ Genomic DNA Mini Kit (Thermo Fisher Scientific). Preparations for the genomic DNA library were constructed using the NEBNext Ultra II DNA Library prep kit (New England Biolabs) according to the manufacturer standard protocol.

### Computational analysis

The sequence reads of DT40 genome were mapped using a Bowtie 2^39^. Mapped data was parsed using samtools^40^.

## Supplementary Information

Supplementary Information.

## Data Availability

Sequencing data were deposited in the DDBJ Sequence Read Archive database under accession numbers DRA012431.
