# Supplementary material for: Targeting chromosome trisomy for chromosome editing
Source: Sci Rep. 2021 Sep 10;11:18054. doi: 10.1038/s41598-021-97580-1 (PMC8433146; doi:10.1038/s41598-021-97580-1)
Supplement: Supplementary file 1 — Supplementary Information. [file 41598_2021_97580_MOESM1_ESM.pdf]

Supplementary Figure 1

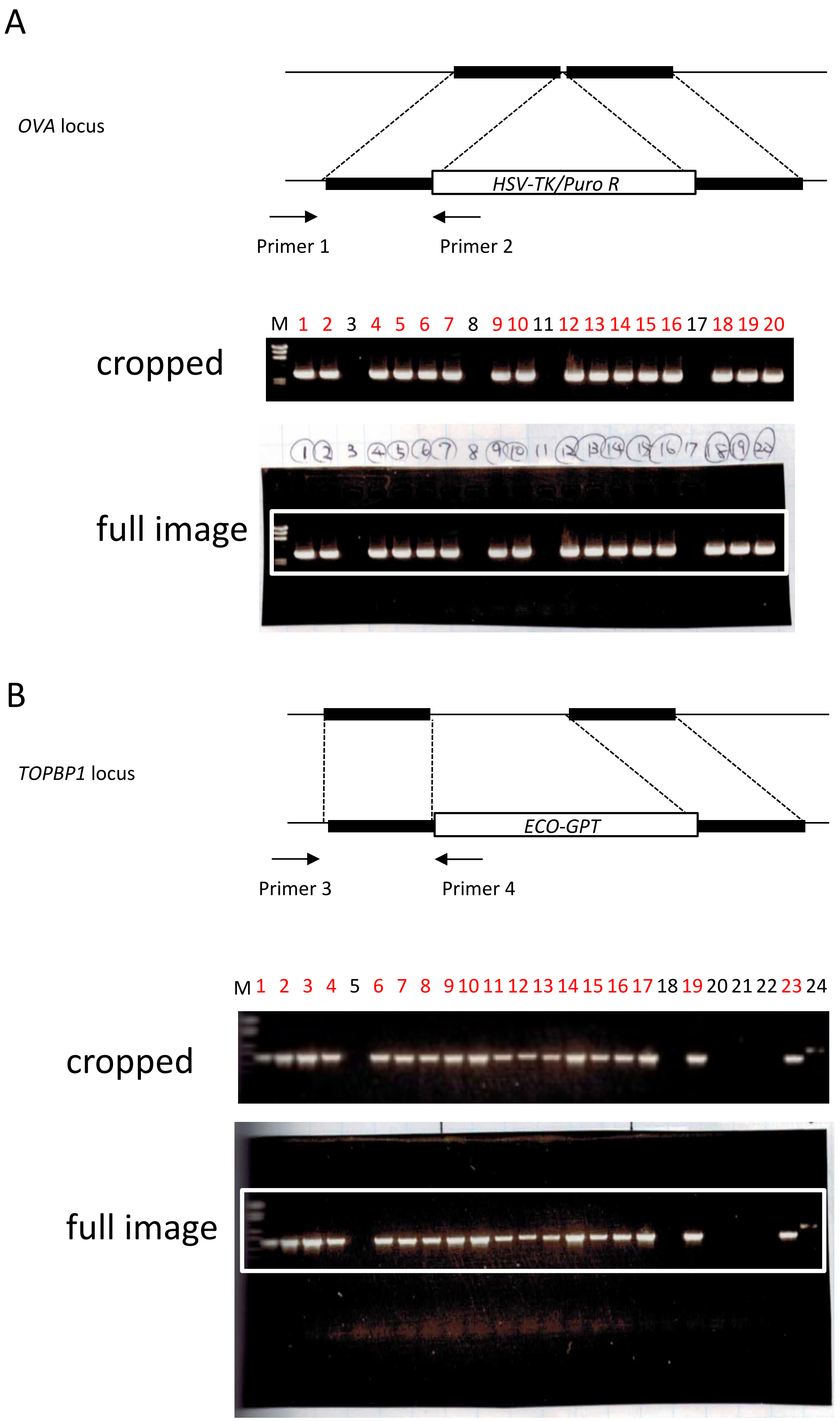

Supplementary Figure 1 Schematic representation of the loci and vectors

Schematic representation of chicken *OVA* (A) and *TOPBP1* (B) loci and the position of primers used to check the targeted integration of the vector. Filled box indicates homology region between the locus and the corresponding vector. The targeted integration is confirmed by PCR below.

Supplementary Figure 2

A

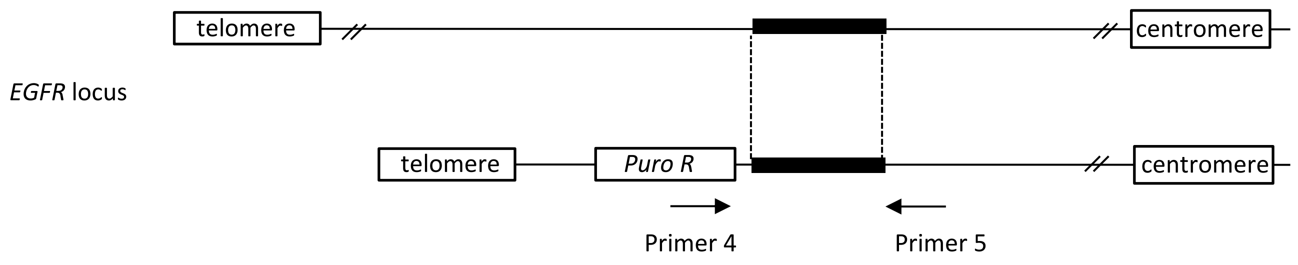

cropped

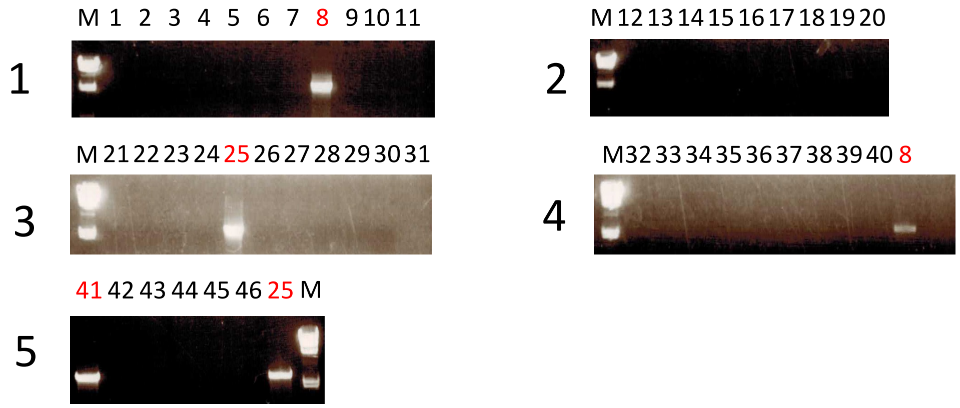

full image

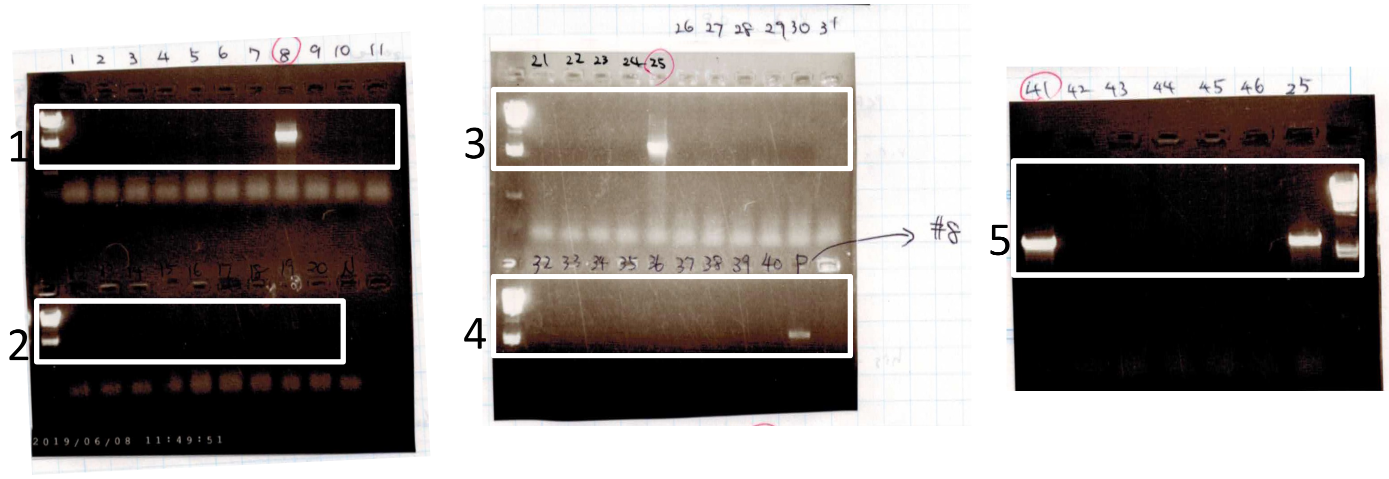

Supplementary Figure 2

B

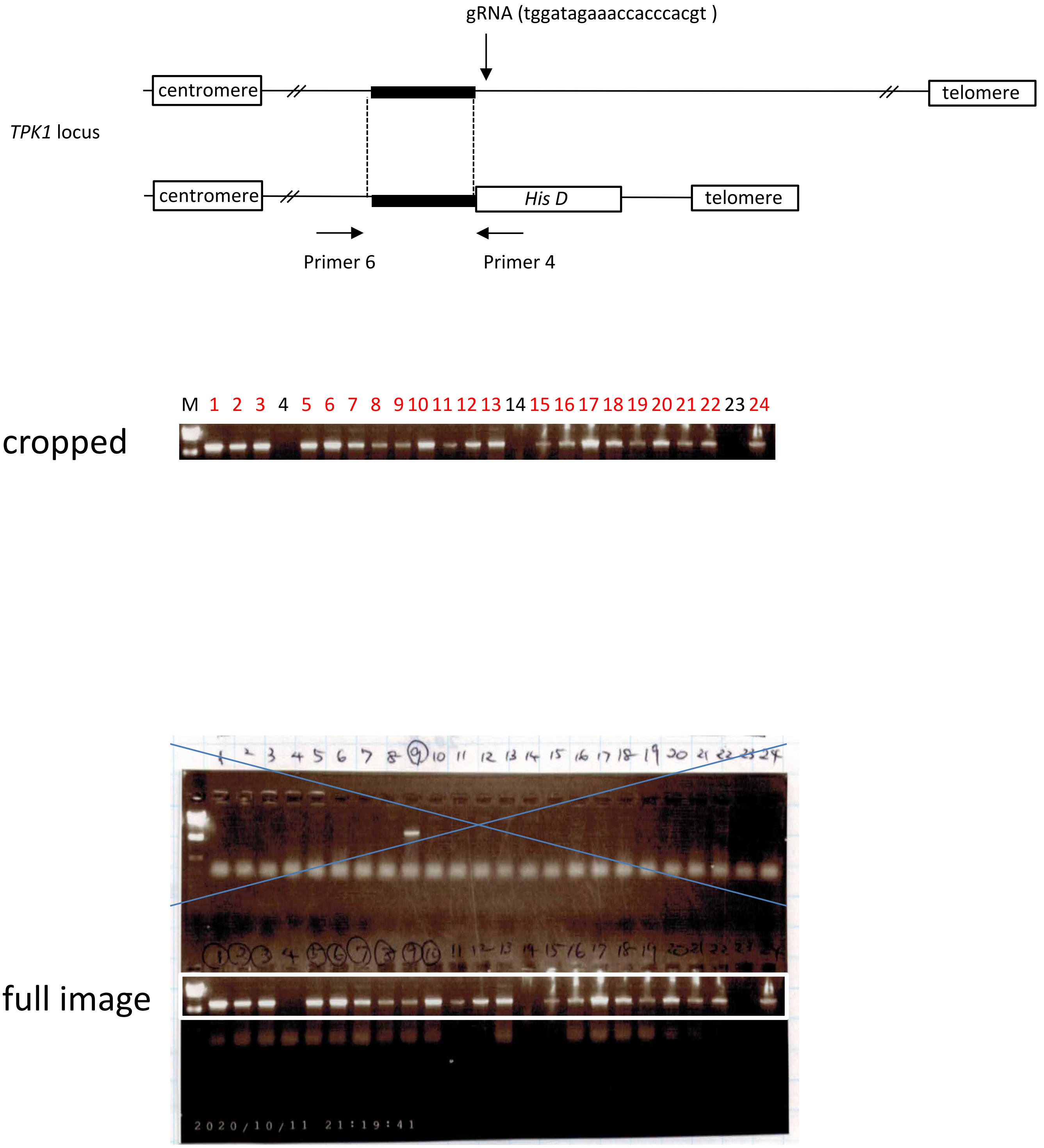

Supplementary Figure 2 Schematic representation of the loci and vectors

Schematic representation of chicken *EGFR* (A) and *TPK1* (B) loci and the position of primers used to check the targeted integration of the vector. Filled box indicates homology region between the locus and the corresponding vector. The targeted integration is confirmed by PCR below.

Supplementary Figure 3

A

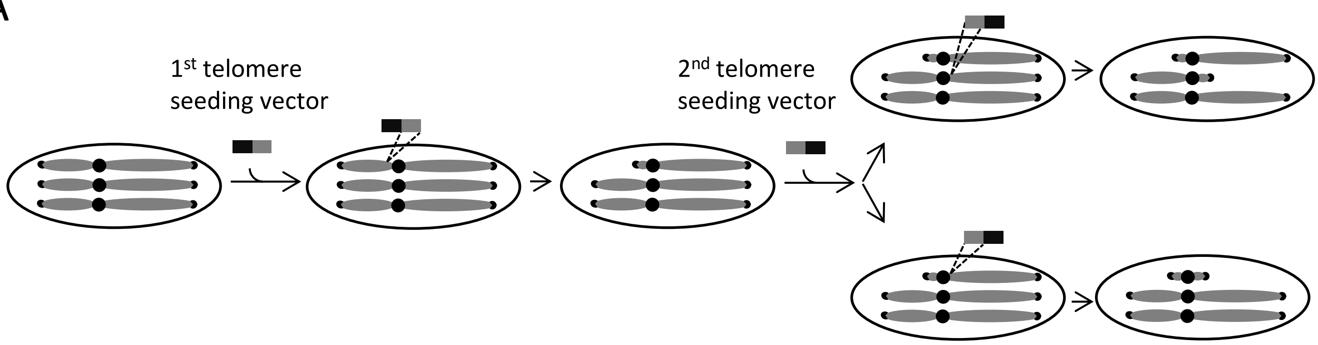

B

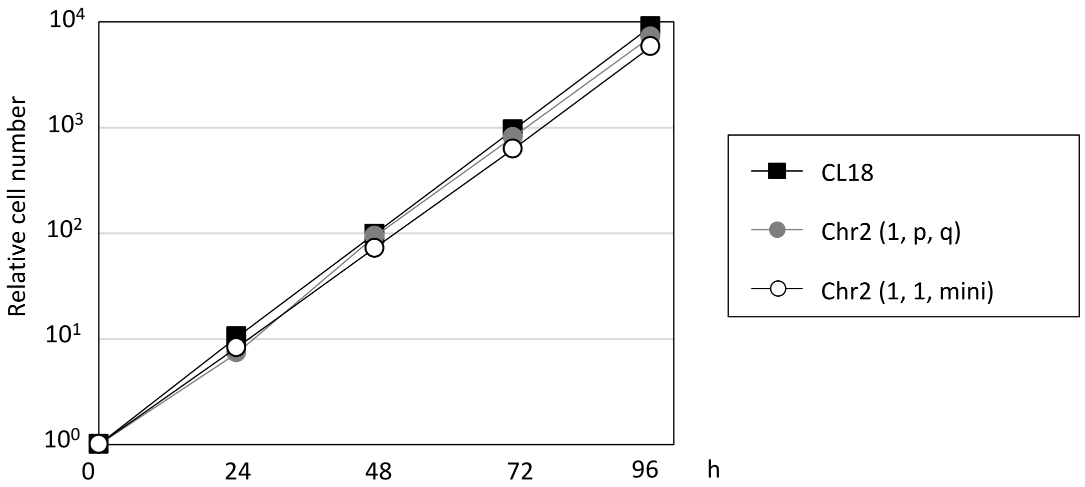

Supplementary Figure 3 Strategy of mini-chromosome development from chromosome 2

(A) Scheme of establishing DT40 Chr2 (1, 1, mini) and Chr2 (1, p, q) cells. (B) Growth curves of DT40 Chr2 (1, p, q) and DT40 Chr2 (1, 1, mini) cells. Cell number was counted and passaged every 24 h using 10^5 cells inoculated in 1 mL of medium.

Supplementary Figure 4

A

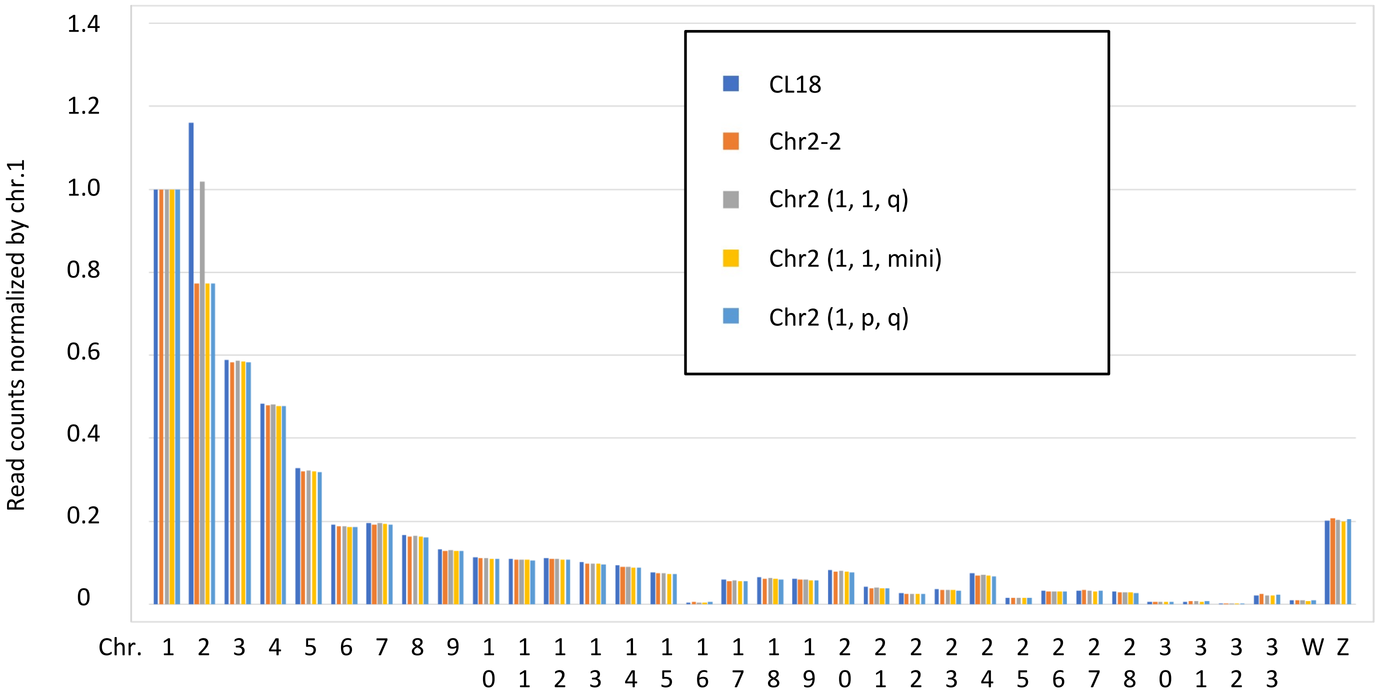

B

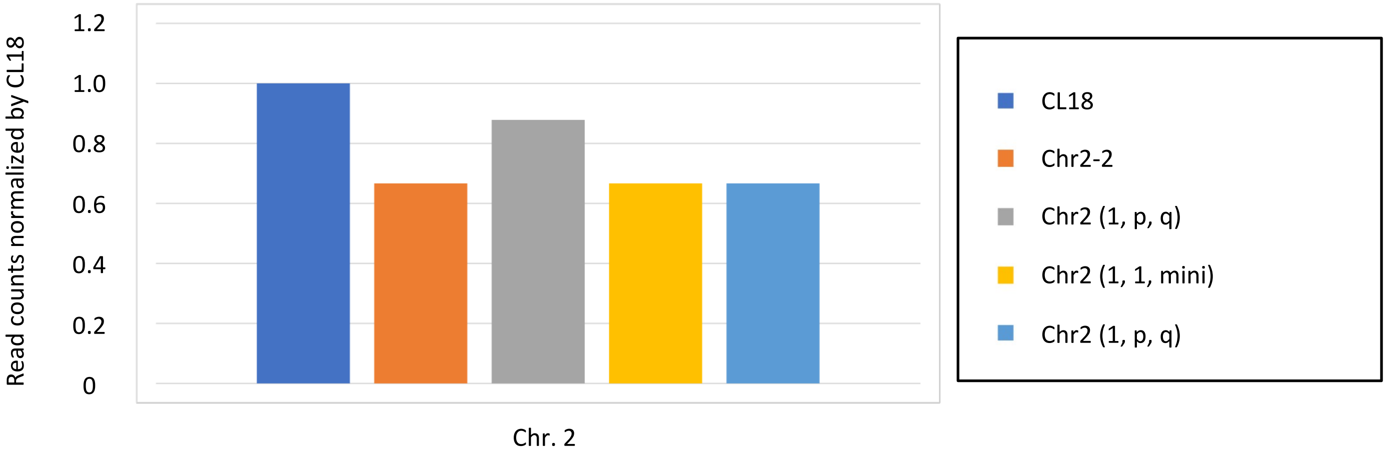

Supplementary Figure 4 Whole chromosome copy number variation

(A) The number of sequence reads mapped on each chromosome was quantified and normalized by those of chromosome 1. (B) The number of sequence reads mapped on chromosome 2 was normalized by those of chromosome 1. This score of each strain was further normalized by that of CL18 cells.

Supplementary Figure 5

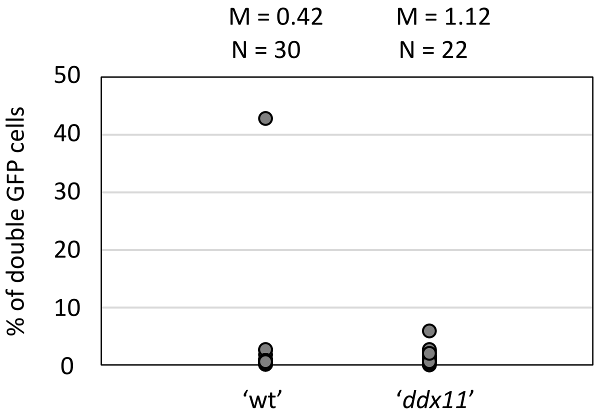

Supplementary Figure 5 Quantification of cells with two mini-chromosomes

DT40 Chr2 (1, 1, mini-GFP) cells (shown as ‘wt’) and *DDX11*$^{-/-}$ Chr2 (1, 1, mini-GFP) cells (shown as ‘*ddx11*’) were preincubated with puromycin for 24 h. Then, puromycin was washed out, and the cells were inoculated in 96-well plates by limiting dilution. Each clone from single cell was analyzed by flow cytometer 7 days after the limiting dilution as performed in Fig. 4A. The percentages of double GFP cells were plotted.

Supplementary Figure 6

cropped

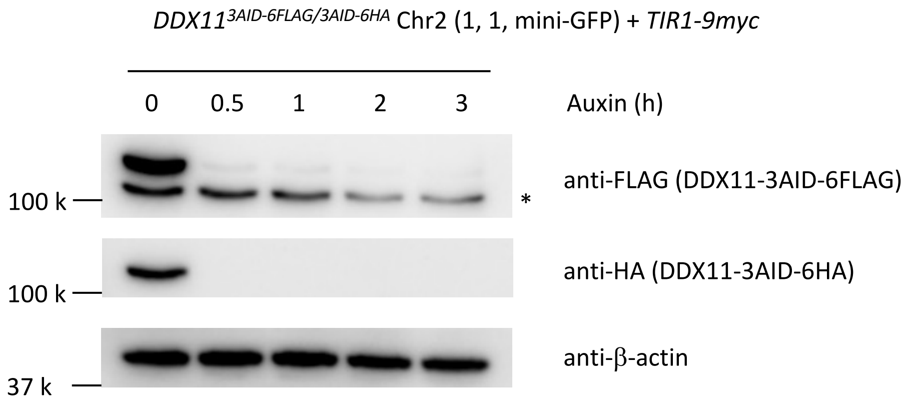

full image

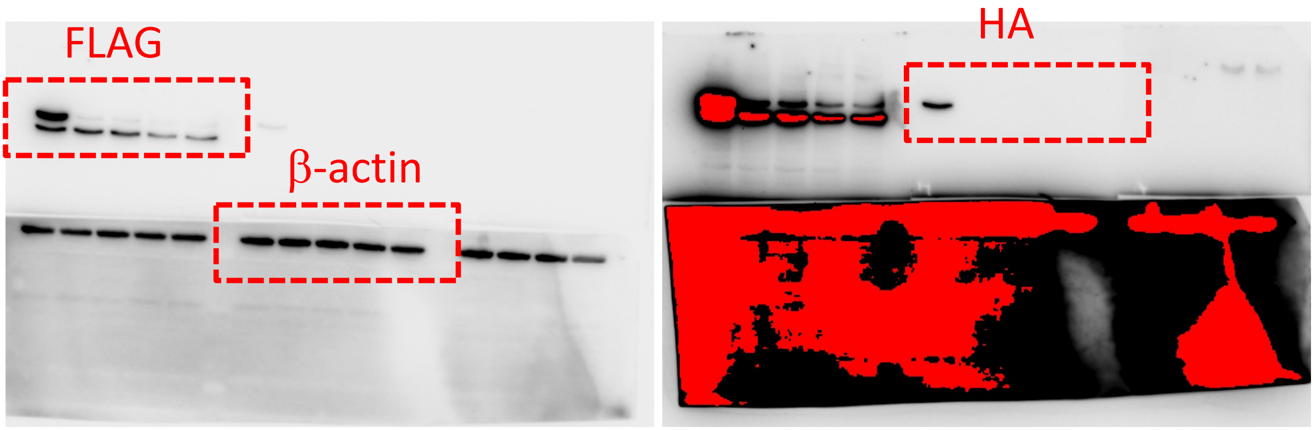

Supplementary Figure 6 Validation of DDX11 depletion

DDX11$^{3AID\text{-}6FLAG/3AID\text{-}6HA}$ Chr2 (1, 1, mini-GFP) + *TIR1-9myc* cells were incubated with 500 μM of auxin for indicated times. The whole cell lysates were used for western blotting. * indicates non-specific bands.

Supplementary Figure 7

A low exposure

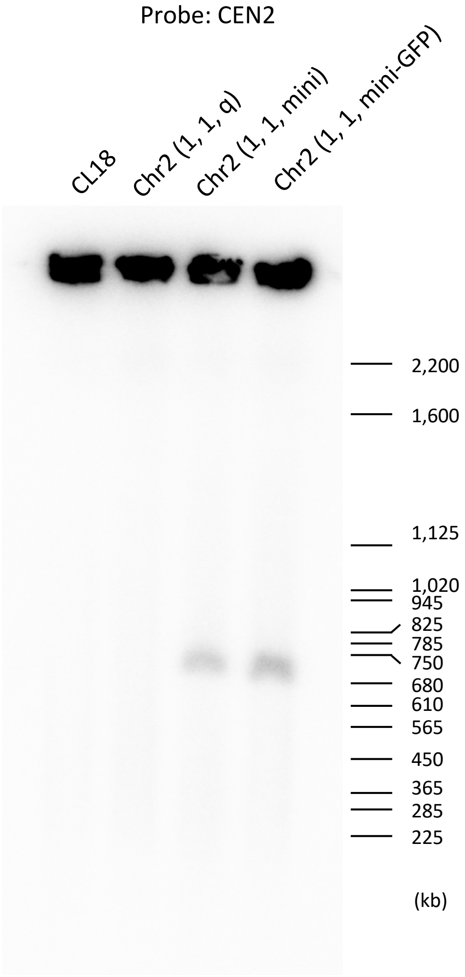

Supplementary Figure 7

B low exposure

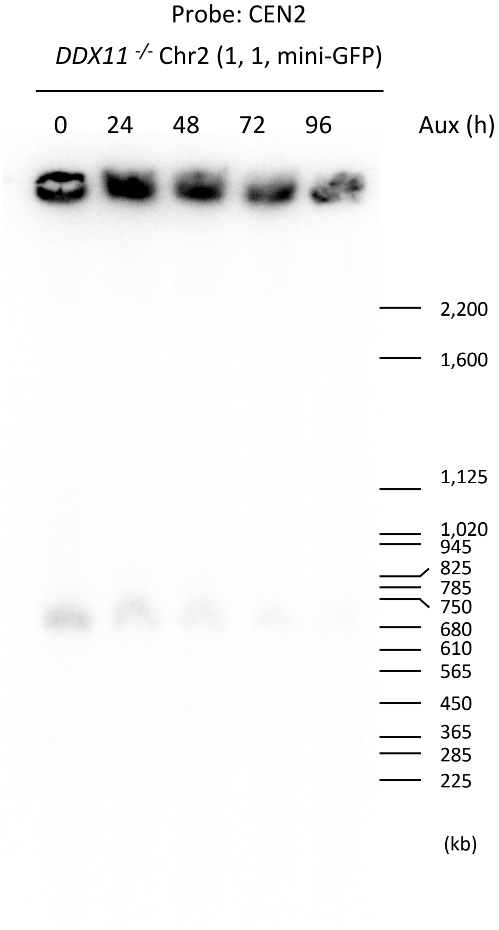

Supplementary Figure 7

C

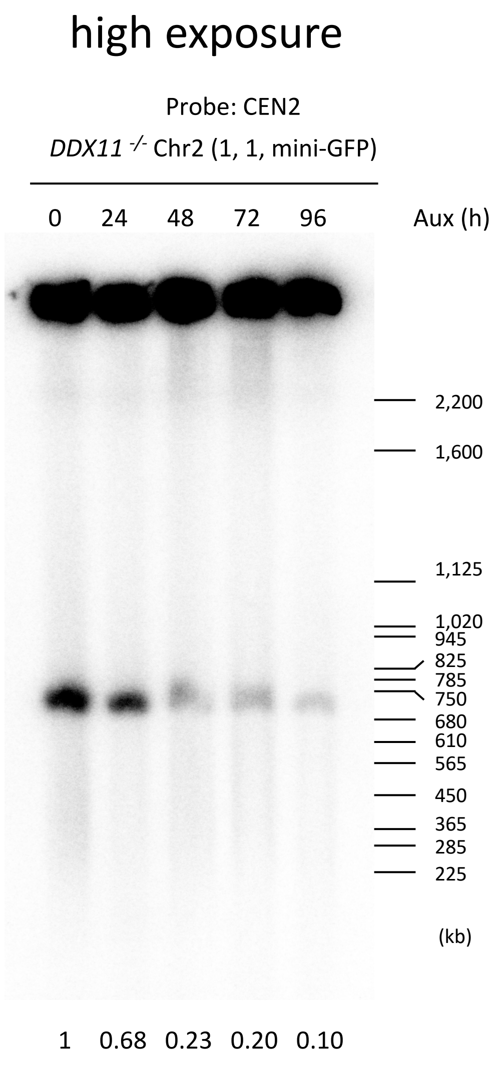

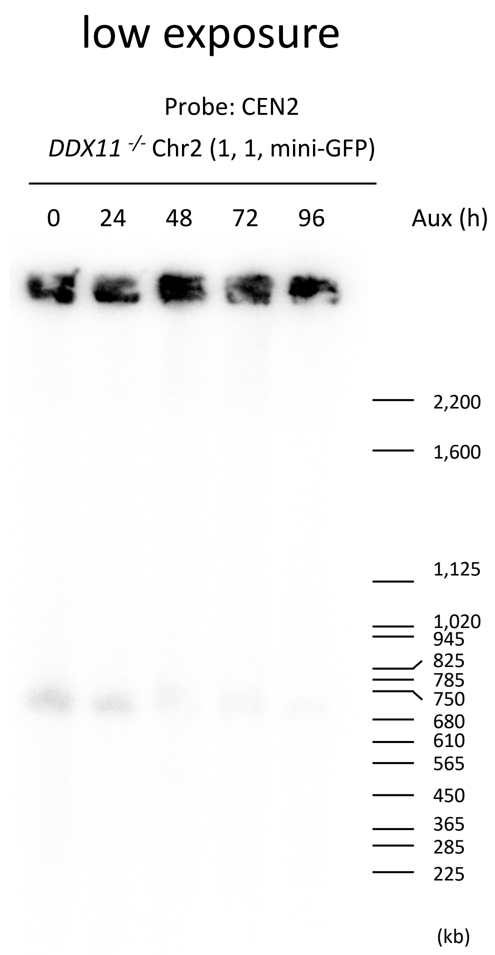

Supplementary Figure 7 PFGE analysis to detect mini-chromosome loss

(A) Low exposure image corresponding to Figure 6C was shown. (B) Low exposure image corresponding to Figure 6D was shown. (C) PFGE analysis was performed to detect chromosome 2 driven mini-chromosome. To show the reproducibility of the result shown in Figure 6D, a result from another experiment was shown. The intensity of band corresponding to mini-chromosome was normalized by that in wells. The scores below the image show the ratios to time 0.
